# Efficacy and prognosis of neuroendoscopy-assisted surgery for chronic subdural hematoma

**DOI:** 10.12669/pjms.39.2.6642

**Published:** 2023

**Authors:** Baolin Ma, Huping Song, Wuyong Lin

**Affiliations:** 1 Baolin Ma, Department of Neurosurgery, The First Affiliated Hospital of Xiamen University, Xiamen, Fujian, 361022, China; 2 Huping Song, Department of Neurosurgery, The First Affiliated Hospital of Xiamen University, Xiamen, Fujian, 361022, China; 3 Wuyong Lin, Department of Neurosurgery, The First Affiliated Hospital of Xiamen University, Xiamen, Fujian, 361022, China

**Keywords:** Chronic subdural hematoma, Neuroendoscopy, Borehole drainage, Hematoma removal

## Abstract

**Objective:**

This paper aims to analyze the clinical effect and prognosis of neuroendoscopy-assisted surgery for chronic subdural hematoma (CSDH).

**Methods:**

The clinical data of CSDH patients who underwent surgery between March 2018 and June 2020 in the Department of Neurosurgery of the First Affiliated Hospital of Xiamen University were retrospectively collected and analyzed. Eighty patients with CSDH who met the inclusive criteria were selected. A control group (32 cases treated with burr hole drainage) and an observation group (48 cases treated with neuroendoscopy-assisted surgery) were set according to different operation methods. The hematoma clearance rate, surgery-related indicators, related complications, hematoma recurrence rate and related prognostic indicators of the two groups were compared and analyzed.

**Result:**

The postoperative hematoma clearance rate of the observation group was 92.59%, which was higher than that of the control group (77.78%) (P<0.05). The operation time of the observation group was longer than that of the control group (P<0.05). The postoperative hospitalization time of the observation group was shorter than that of the control group (P<0.05). The postoperative complication rate of the observation group was lower than that of the control group (P<0.05). The recurrence rate of hematoma in the observation group in the six-month postoperative follow-up was 1.85%, which was lower than that in the control group (P<0.05). The limb motor function and daily living ability score of the observation group were higher than those of the control group, and the Markwalder grading score was lower than that of the control group (P<0.05).

**Conclusion:**

Neuroendoscopy-assisted treatment which is safe and effective is superior to traditional burr-hole drainage surgery. It can reduce the recurrence rate; thus, it is worth advocating and applying.

## INTRODUCTION

Chronic subdural hematoma (CSDH) is a common neurosurgical disease that has been clinically shown to account for approximately 10% of all intracranial hematomas.^1^ CSDH means that subdural hematoma shows clinical symptoms such as seizures, psychiatric abnormalities, chronic increased intracranial pressure and abnormal limb movements three weeks after trauma, which has a high incidence.^2^,^3^ Yang et al. reported an annual incidence of CSDH ranging from 1.72% to 20.6% per 100,000 people.^4^ CSDH has a slow onset and is usually ignored or misdiagnosed because of lacking first symptoms, but with the development of imaging technology, cranial examination by computed tomography (CT) and magnetic resonance imaging (MRI) can confirm the diagnosis in the majority of cases. To date, the specific pathogenesis of CSDH has not been fully elucidated. CSDH is often accompanied by neurological impairment symptoms, such as intracranial hypertension, memory loss and progressive dementia. Seriou’s sequelae will be left if timely and effective treatment is not provided for CSDH, affecting the patient’s ability to perform daily activities and neurological functions.

Surgery is currently the common clinical treatment for CSDH, including single hole drainage, double borehole drainage and minimally invasive puncture drainage, but clinical practice shows that borehole drainage will cause severe trauma, which is difficult to meet the increasing needs of patients.^5^,^6^

In recent years, the application of neuroendoscopy in the treatment of CSDH has been increasingly reported. Through neuroendoscopy, the entire hematoma cavity can be visualized to completely remove hematoma and trabecular-like structure and adequately stop bleeding, and a hematoma cavity drain tube can be placed under direct visualization.^7^ The recurrence of postoperative hematoma is an important indicator to assess the efficacy of CSDH, and the recurrence rate was reported to be 10%-20%.^8^ The recurrent hematoma pushes and compresses the brain tissue, causing irreversible damage to the already atrophied brain tissue and seriously affecting the quality of life and prognosis of patients. Guo et al. reported a recurrence rate of 5.3% in the neuroendoscopy-assisted treatment of 187 cases of CSDH.^9^ Another study compared endoscope-assisted burr-hole craniostomy and ordinary burr-hole craniostomy in the treatment of septated CSDH.^10^ The results showed that the recurrence rate of endoscope-assisted burr-hole craniostomy was low, there was no significant difference in the complications between the two procedures, and the effect of endoscope-assisted burr-hole craniostomy was superior to that of ordinary burr-hole craniostomy. Currently, there is no uniform opinion on the surgical method for patients with CSDH. In order to improve the clinical treatment effect of patients with CSDH and reduce the postoperative complications and recurrence rate, this study statistically analyzed the relevant clinical data of 80 CSDH patients who underwent surgery between March 2018 and June 2020 in the Department of Neurosurgery of the First Affiliated Hospital of Xiamen University.

## METHODS

In this study, 80 CSDH patients who were admitted to the First Affiliated Hospital of Xiamen University. between March 2018 and June 2020 were selected for retrospective analysis.

### Inclusion criteria:

- Time since onset > three weeks, having clinical symptoms such as headache, dizziness and limb function limitation;
- Diagnosed as chronic subdural hematoma by cranial scan, with significant midline structural displacement of unilateral hematoma and insignificant midline displacement of bilateral hematoma;
- Volunteer to receive hematoma burr hole drainage and have signed the relevant informed consent.

### Exclusion criteria:

- Having abnormal coagulation function;
- Unable to tolerate local and general anesthesia;
- Having received conservative treatment or cranial hematoma removal;
- Having incomplete clinical data and lost to follow up in the middle.

Thirty-two patients who received burr hole drainage were taken as the control group, and 48 patients were included as the observation group. In the control group, there were 27 males and five females; their age ranged from 49 to 74 years, with a mean age of (63.58±2.93) years; the hematoma volume ranged from 40.45 to 90.30 mL, with a mean hematoma volume of (81.25±4.94) mL. In the observation group, there were 40 males and eight females; their age ranged from 43 to 76 years, with a mean age of (64.12±7.40) years; the hematoma volume ranged from 42.33 to 91.11 mL. The differences between the two groups in the general data were not statistically significant (P>0.05); thus, the results were comparable. The study was approved by the medical ethics committee of the First Affiliated Hospital of Xiamen University (No. 2021108 dated on July 25^th^ 2021).

The patient underwent local anesthesia. According to the cranial CT or MRI examination, a straight incision of about 5cm was cut longitudinally, centered on the thickest level of the hematoma. The skull was drilled, and the diameter of the bone hole was enlarged to about 2cm. Then, a cross-shaped incision was cut on the dura mater, and a silicone drainage tube was inserted. The hematoma fluid was flushed repeatedly until the flushing fluid became clean. The silicone tube was left in the hematoma cavity and properly fixed, and the incision was sewed up.

The patient took a supine position under assistance. The affected shoulder was elevated, and the head tilted 30°-60° to the healthy side. Then, general anesthesia was administered by tracheal intubation. In the positioning process, taking the thickest level of the hematoma strictly based on the preoperative CT or MRI examination results as the center, a straight incision or inverted U-shaped incision was cut. After the flap was cut using a milling cutter and cranial drill, a 4.0 cm × 4.0 cm bone window was opened. The tissues around the dura mater were suspended and cut apart. After the removal and electrocoagulation of partial hematoma and envelope under direct vision. A neuroendoscope (manufacturer: Geman Philipp, model number: Bozzini) was inserted. Multiple hematoma fibroblast-like cord septa were removed by electric-coagulation and division under the neuroendoscope to prevent intraoperative blood loss. A large amount of saline was used to ensure complete removal of the hematoma in the multiple septa.

Patients in the two groups were followed up by telephone and reexamined regularly. The following indicators were observed. (1) Hematoma clearance rate (hematoma-free one week after surgery). (2) Operative time, postoperative hospitalization time, and hospitalization cost. (3) Incidence of complications one week after surgery and recurrence rate of hematoma in six-month follow-up. (4) Neurological function. Markwalder grading scoring method was used one week after surgery. It divides neurological function into four levels. No neurological deficits were evaluated as Level-0 (0 point); mild neurological deficits such as mild headache and asymmetry of tendon reflexes were evaluated as Level-1 (1 point); severe neurological deficits such as disorientation, drowsiness and mild hemiparesis were evaluated as Level-2 (2 points); severe neurological deficits such as shallow coma, catalepsy and hemiparesis were graded as Level-3 (3 points); extremely severe neurological deficits such as unresponsive to painful stimuli, coma, decortication, and decerebrate rigidity were evaluated as Level-4 (4 points).^11^ (5) Limb motor function and daily living ability. The limb motor function and daily living ability were evaluated by the Fugl-Meyer assessment (FMA) scale^12^ and Barthel Index (BI)^13^ one week after surgery. The FMA scale includes limb movement, sensation, pain, etc., and the total score was 0-100 points. The higher the FMA scale score is, the better the limb motor function is. The total score of BI was 0~ 100 points. The higher the FMA scale score was, the stronger the daily living ability was.

### Statistical Analysis:

SPSS 20.0 statistical software was used to analyze the data. The measurement data were expressed as () and processed by t-test. The count data were expressed as percentages and processed by X2 test. P<0.05 indicated that the difference was statistically significant.

## RESULTS

The postoperative hematoma clearance rate was 91.67% (44/48) in the observation group, which was higher than that of 75.00% (24/32) in the control group. The difference was statistically significant (X^2^=16.217, P<0.05).

The operation time was longer in the observation group than in the control group (P<0.05). The postoperative hospital stay was shorter in the observation group than in the control group (P<0.05). There was no statistically significant difference between the two groups in terms of hospitalization costs (P>0.05) (Table-I).

**Table-I:** Comparison of surgical indicators between the two groups (*χ̅*±*s*)

Group	Observation group	Control group	t	P
Operation time (min)	104.88±28.21	65.20±21.65	7.104	<0.05
Postoperative hospital stay (d)	8.46±2.35	10.22±2.03	3.535	<0.05
Hospitalization cost (ten thousand)	2.04±0.25	2.05±0.36	0.297	>0.05

There were no surgery-related complications in the observation group. There were two cases of motor aphasia and two cases of hemiparesis in the control group. The incidence of postoperative complications was 0 in the observation group, which was lower than that of 12.50% (4/32) in the conventional group (X^2^=3.849, P<0.05). The recurrence rate of hematoma in the observation group was 2.08% (1/48) in the six-month follow-up, which was lower than 15.63% in the control group (X^2^=3.165, P<0.05).

Before treatment, there was no statistically significant difference in the Markwalder grading score, limb motor function score and daily living ability score between the two groups (P>0.05). After treatment, the Markwalder grading score of the observation group was lower than that of the control group, and the limb motor function and daily living ability scores were higher than those of the control group (P<0.05) (Table-II).

**Fig.1:**
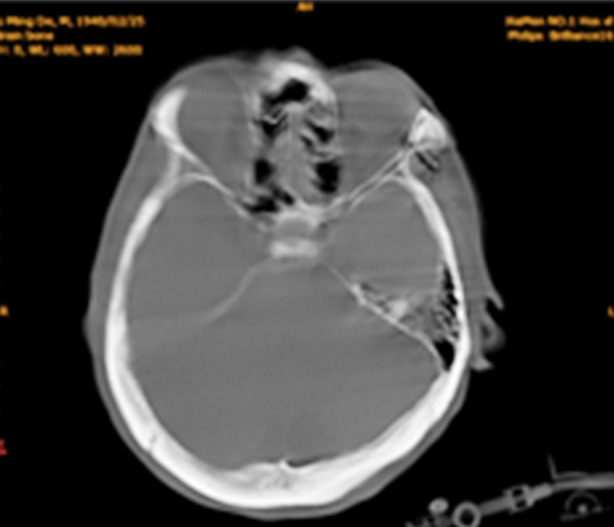
CT manifestations of CSDH

**Table II:** Comparison of neurological function, limb motor function, and daily living ability between the two groups [score, (*χ̅*±*s*)].

Group		Observation group	Control group	t	P
Markwalder grading score	Before treatment	2.28±0.39	2.31±0.31	0.134	>0.05
	After treatment	0.72±0.12	1.51±0.11	30.214	<0.05
FMA scale score	Before treatment	56.65±9.44	55.47±9.45	0.496	>0.05
	After treatment	87.64±9.16	71.25±9.45	8.357	<0.05
BI score	Before treatment	53.66±8.45	54.25±9.23	0.317	>0.05
	After treatment	85.35±9.26	69.46±9.45	8.102	<0.05

## DISCUSSION

Relevant medical studies have shown that neuroendoscopy-assisted surgery has good effect and high reliability and safety in the treatment of septated CSDH and can be the preferred clinical procedure.^14^-^16^ In this study, 48 patients underwent neuroendoscopy-assisted surgery. The liquefied hematoma flowed out after opening the dura mater and wall envelope intraoperatively, and the neuroendoscopic exploration revealed that the residual blood clots in the hematoma cavity were deposited in the visceral envelope or in the interstitial space at the envelope reflection, which could be easily removed by suction. There was no obvious adhesions and no dense separation cavity, indicating that the hematoma could be adequately flushed through the small bone window. Intraoperatively, it was seen that trabecular structures connected the visceral envelop and the wall envelop. Takei et al. reported that some of the trabecular structures contained blood vessels and might be involved in the formation of CSDH.^17^ We cut trabecular structures off after electrocoagulation and found that the postoperative hematoma clearance rate was higher in the observation group than the control group, which is seldom reported in previous studies. The above results provide a reference for the formulation mechanism of chronic subdural hematoma and verify the effectiveness of neuroendoscopy-assisted surgery, which was consistent with the results of Zhu et al.^18^

The results of this study showed that the operation time was longer in the observation group than in the control group, but the postoperative hospital stay and postoperative complication rate were better in the observation group than in the control group (P<0.05). The long duration of neuroendoscopy-assisted surgery is mainly related to its relatively difficult operation and the need for multiple cuts and separations during the procedure.^19^ However, the greatest advantage of neuroendoscopic assistance is that it can provide clinicians with a better operative field, thus increasing the precision. In addition, neuroendoscopic assistance has electrocoagulation and suction working channels that allow direct hematoma suction.^20^ Unlike simple drilling and drainage, neuroendoscopy can clearly reflect hematoma separation. Under neuroendoscopy, hematoma separation can be accurately disrupted and removed, reducing inflammatory reactions.

In addition, the recurrence rate of hematoma in the observation group was lower than that in the control group in the six-month follow-up (P<0.05). The surgical procedure in the control group is the current conventional clinical treatment method, which is simple to operate, remits symptoms quickly, and causes little damage to the patient, but there is still a recurrence rate of 3.7%-38% after surgery.^21^ Neuroendoscopy can observe the septa of the hematoma cavity and assist in the removal of septa by bipolar electrocoagulation, which facilitates the reduction of subdural fluid and greatly reduces the probability of recurrence. In addition, the scores of limb motor function and daily living ability in the observation group were higher than those in the control group, suggesting that neuroendoscopy-assisted surgery could improve patients’ neurological function and daily living ability, and the finding was in line with the research results of Chen et al.^22^

### Limitations:

The small sample size in this study may lead to bias in the data results, and the relevant surgical indicators collected were not complete, such as the amount of subdural fluid and surgical bleeding, which will be studied in the future.

## CONCLUSION

Neuroendoscopy-assisted surgery has a good clinical effect, a low recurrence rate, and a good prognosis in treating CSDH.

### Authors’ Contribution:

**BLM:** Study design, data collection and analysis.

**BLM, HPS & WYL:** Manuscript preparation, drafting and revising.

**WYL:** Review and final approval of manuscript; He is also responsible and accountable for the accuracy or integrity of the work.
